# Cesium Modulation in Cu(In, Ga)(S, Se)_2_ Solar Cells: Comprehensive Analysis on Interface, Surface, and Grain Boundary

**DOI:** 10.1021/acsami.4c03680

**Published:** 2024-06-12

**Authors:** Yung-Hsuan Chen, Rui-Tung Kuo, Wei-Chih Lin, Chien-Yu Lai, Tzu-Ying Lin

**Affiliations:** Department of Materials Science and Engineering, National Tsing Hua University, 101, Sec. 2, Kuang-Fu Road, Hsinchu 300044, Taiwan

**Keywords:** Cu(In,Ga)(S,Se)_2_, cesium, grain boundary, bandgap widening, LEIPS

## Abstract

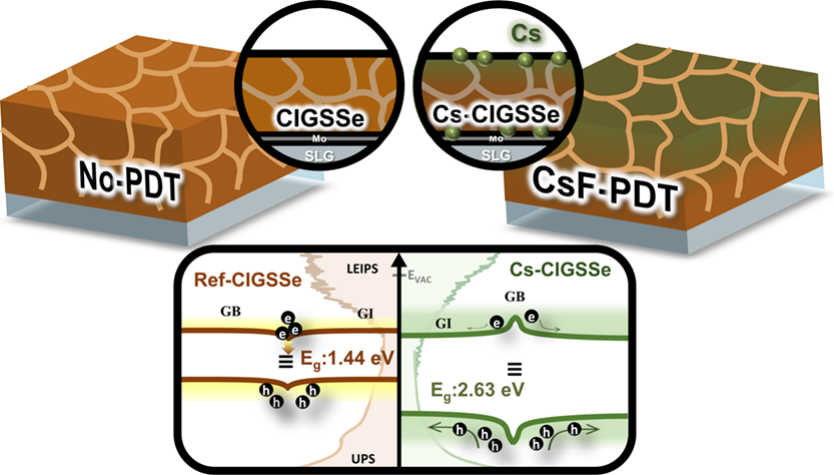

Cesium (Cs) incorporation and sulfurization on copper indium gallium selenide solar cells are the keys to improving the device quality. In this study, we explore the impact of Cs modulation on sulfur-containing Cu(In, Ga)(S, Se)_2_ (CIGSSe) absorbers, resulting in a performance increase of over 2%, reaching 18.11%. The improvement stems from a widened surface bandgap, grain boundary (GB) passivation, and a moderate injection blocking layer. The surface bandgap widens from 1.44 to 2.63 eV after Cs incorporation, confirmed by ultraviolet photoelectron spectroscopy (UPS) and low-energy inverse photoemission spectroscopy (LEIPS) analysis. Cs presence and S depletion in GBs suggest a new phase that might mitigate carrier recombination. Heightened Cs incorporation introduces interface issues, including an augmented injection blocking layer and interface defects. Our study offers insights into interface challenges and GB engineering strategies in Cs-treated CIGSSe solar cells, illuminating the multifaceted impact of heavy alkali metal ion Cs in CIGS-based photovoltaics.

## Introduction

Chalcopyrite Cu(In,Ga)Se_2_-based thin-film solar cells have been some of the most promising thin-film solar cells. This is primarily due to its high absorption coefficient, direct and tunable bandgap as well as its long-term stability.^1^ In addition, CIGS-based solar cells have demonstrated potential for various applications, including portable devices, aerospace, and building-integrated photovoltaics.^2−5^

In reviewing the progress of CIGS solar cells, incorporating alkali metal into CIGS-based solar cells is the striking point of enhancing the power conversion efficiency (PCE). CIGS-based solar cells have achieved a PCE of over 23%.^6,7^ The introduction of Na into the CIGS absorber plays a crucial role in increasing the net hole concentration by passivating deep donor-like defects, such as In_Cu_ and Ga_Cu_, thereby enhancing p-type conductivity.^8,9^ Additionally, the incorporation of heavier alkali metal ions via postdeposition treatment (PDT) such as K,^10,11^ Rb,^12,13^ and Cs^14^ has also been observed to further improve the absorber quality.

Numerous research studies have delved into the modification of CIGS-based solar cells following alkali-PDT. The studies have reported the depletion of Cu and Ga near the front interface in alkali-PDT devices.^11,14,15^ The ion exchange effect of heavier alkali metal ions on the CIGS-based solar cells with lighter alkali metal ions has also been observed.^13,14^ Owing to the compatibility of atomic radius, lighter alkali metal ions tend to occupy the Cu sites, while heavier alkali metal ions tend to stay at the grain boundary.^14,16−19^ In addition, the formation of high bandgap alkali–In-Se-related compounds may be considered as the passivation layer at the p–n junction. Nevertheless, the existence of the high bandgap alkali–In-Se-related compound following alkali-PDT strongly depends on the composition of the CIGS absorber or alkali incorporation degree, which is not universally observed on all alkali-PDT devices.^20−23^ Therefore, the promising reasons for this efficiency boost are still the subject of controversy. Furthermore, limited research exists on CIGS absorbers further sulfurized, specifically Cu(In, Ga)(S, Se)_2_ (CIGSSe) with CsF-PDT, despite its potential to enhance device performance. The incorporation of Cs in CIGSSe solar cells has sparked interest in elucidating its mechanisms, particularly since achieving the first record efficiency exceeding 23% in CIGS-based solar cells with Cs-incorporated CIGSSe.^6^

In this study, we first studied the device properties with Cs incorporation on S-containing CIGSSe. Different amounts of Cs were incorporated to investigate the effects of Cs on the CIGSSe absorber. Illumination- and temperature-dependent characterizations using Suns-*V*_OC_ and *V*_OC_-T measurements were carried out to estimate the recombination behaviors and assess the quality in interface, depletion, and bulk. Besides, capacitance spectroscopy was also utilized to evaluate the blocking layer thickness and estimate the defects introduced after Cs incorporation. Bias-assisted admittance spectroscopy was further applied to characterize the uniformity of the defect distribution. After the interface study, we delved into the characterization of the surface and grain boundaries (GBs). The status of the surface bandgap with Cs incorporation was reconstructed using ultraviolet photoelectron spectroscopy (UPS) and low-energy inverse photoemission spectroscopy (LEIPS). Kelvin probe force microscopy (KPFM) analysis revealed the correlation between the Cs compound and the band bending diagram at GBs. Transmission electron microscopy (TEM) analysis further verifies the composition of the modified grain surface and GBs. Through a thorough investigation of Cs-treated CIGSSe solar cells, we gain more insight into interface issues and strategies for surface and grain boundary engineering. The interaction mechanism between heavy alkali metal ion Cs and the S-containing quinary system can be further elucidated through future studies focusing on the dynamics of ion migration and incorporation. This comprehensive approach sheds light on the multifaceted impact of Cs in CIGS-based solar cells.

## Results and Discussion

### Cu(In, Ga)(S, Se)_2_ Absorbers with Cs Incorporation: Investigating Interfaces

**Figure 1:**
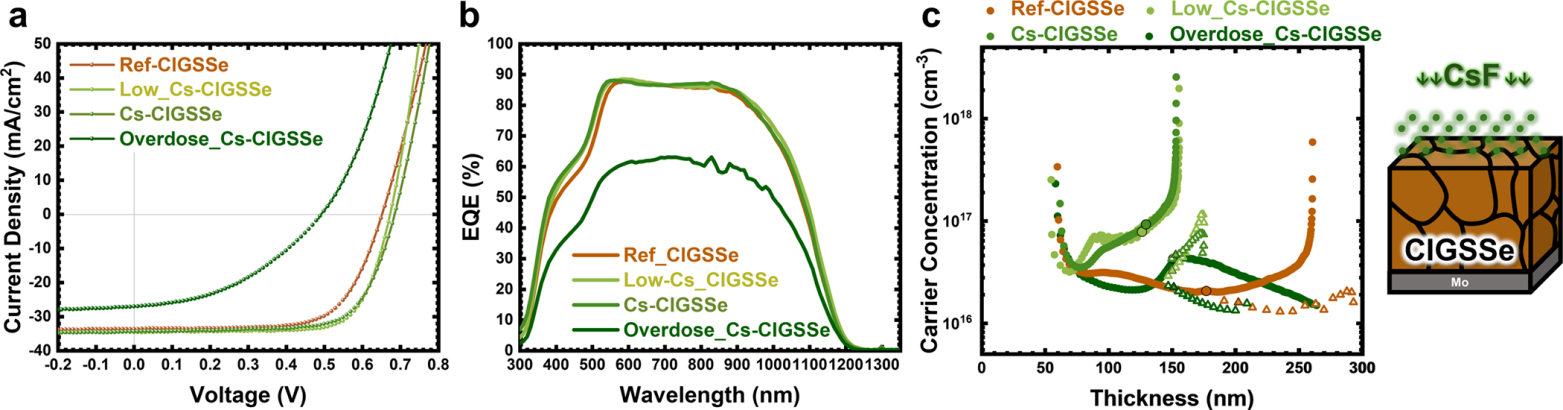
(a) *J–V*, (b) EQE, (c) CV (circle) and DLCP (triangle) characteristics curves of devices without and with CsF-PDT treatment. The black edge of the circles and triangles indicates the measurement under zero bias voltage. The inset figure represents the process of Cs incorporation by CsF-PDT.

To investigate the influence of alkali metal ions, particularly Cs, incorporated into the Cu(In, Ga)(S, Se)_2_ (CIGSSe) absorber, we applied varying levels of CsF-PDT on CIGSSe films produced through the sequential sulfurization after the selenization (SAS) process. Figure 1a shows the device performance with and without Cs incorporation. Detailed values are listed in Table 1. The *V*_oc_ are 0.653 0.672, 0.686, and 0.493 V for devices of ref-CIGSSe, Low_Cs-CIGSSe, Cs-CIGSSe, and Overdose_Cs-CIGSSe, respectively. The *J*_sc_ values are nearly identical in ref-CIGSSe, Low_Cs-CIGSSe, and Cs-CIGSSe. In contrast, substantial current loss is observed across the entire wavelength range only in the case of Overdose_Cs-CIGSSe, as depicted in Figure 1b. Interestingly, the FF, which almost determines the PCEs in this batch, increases in Low_Cs-CIGSSe (FF: 0.74) but gradually decreases in Cs-CIGSSe (FF: 0.70) and Overdose_Cs-CIGSSe (FF: 0.54).

**Table 1:** Summary of the Device Performance of CIGSSe with and without Cs Incorporation^a^

CsF-PDT	*V*_oc_ (V)	*J*_sc_ (mA/cm^2^)	FF	eff.*(*%*)*	*E*_g,min_ (eV*)*	*V*_OC,def_ (eV*)*	*N*_CV_ (cm^–3^)	*W*_CV_ (nm)	*N*_DLCP_ (cm^–3^)	*W*_DLCP_ (nm)
ref-CIGSSe	0.653	33.72	0.68	14.97	1.07	0.417	2.05 × 10^16^	177	1.58 × 10^16^	203
avg.	0.651	33.80	0.67	14.77						
Low_Cs-CIGSSe	0.674	34.64	0.74	17.24	1.08	0.406	7.78 × 10^16^	126	3.23 × 10^16^	151
avg.	0.673	34.48	0.73	17.05						
Cs-CIGSSe	0.686	34.48	0.70	16.68	1.08	0.394	9.20 × 10^16^	130	4.62 × 10^16^	153
avg.	0.684	34.40	0.70	16.38						
Overdose_Cs-CIGSSe	0.514	30.67	0.54	8.48	1.08	0.566	4.23 × 10^16^	151	2.23 × 10^16^	152
avg.	0.512	25.62	0.55	7.15						
Low_Cs-CIGSSe AR	0.677	36.05	0.74	18.11						

a The minimum bandgap (*E*_g, min_) was determined from the absorption edge of the EQE spectrum. AR denotes antireflection coating.

Capacitance–voltage (*C–V*) and drive level capacitance profiling (DLCP) measurements were employed to evaluate the doping profile and variations in carrier concentration, as illustrated in Figures 1c and S1 and summarized in Table 1. The carrier concentration obtained by *C–V* measurement (*N*_CV_) improves from 2.05 × 10^16^ cm^–3^ in ref-CIGSSe to 7.78 *×* 10^16^ cm^–3^ in Low_Cs-CIGSSe, and further to 9.20 *×* 10^16^ cm^–3^ in Cs-CIGSSe. However, it drops to 4.23 *×* 10^16^ cm^–3^ in Overdose_Cs-CIGSSe. On the other hand, the carrier concentration obtained through DLCP measurements (*N*_DL_) (see Table 1, ref-CIGSSe: 1.58 *×* 10^16^ cm^–3^, Low_Cs-CIGSSe: 3.23 *×* 10^16^ cm^–3^, Cs-CIGSSe: 4.62 *×* 10^16^ cm^–3^, and Overdose_Cs-CIGSSe: 2.23 *×* 10^16^ cm^–3^), known for its reduced sensitivity to interface states,^24^ exhibits a consistent trend with *N*_CV_ values. The discrepancies in *N*_CV_ and *N*_DL_ may result from the presence of interface defects. Furthermore, the increase in *V*_OC_ may also be attributed to the enhancement of carrier concentration, as indicated in Table S2.

**Figure 2:**
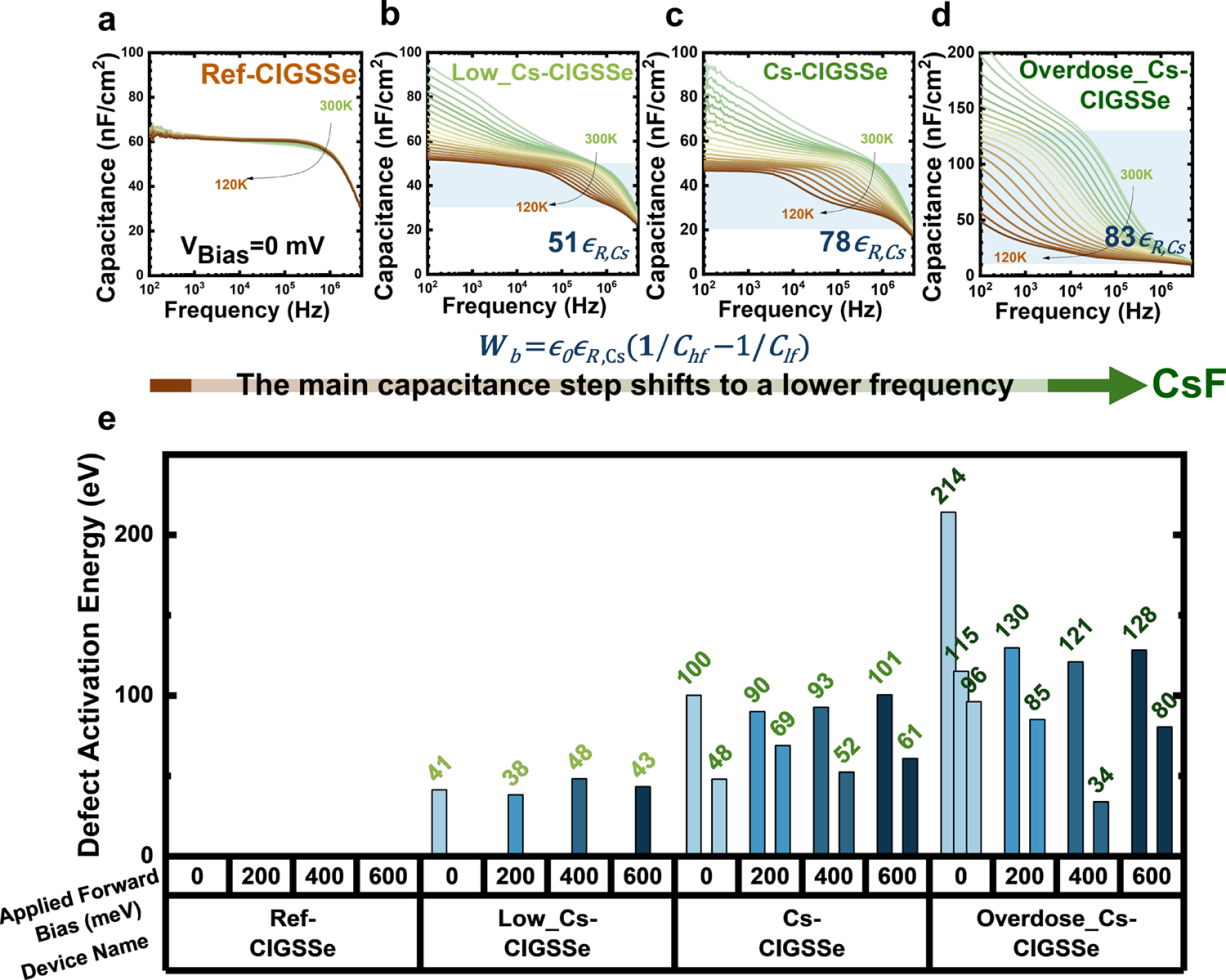
Temperature-dependent admittance capacitance-frequency spectra of the samples with different amounts of Cs. (a) ref-CIGSSe, (b) Low_Cs-CIGSSe, (c) Cs-CIGSSe, and (d) Overdose_Cs-CIGSSe devices under 0 bias. (e) Extracted defect activation energies from admittance spectra under 0, 200, 400, and 600 meV of ref-CIGSSe, Low_Cs-CIGSSe, Cs-CIGSSe, and Overdose_Cs-CIGSSe devices.

For a more thorough assessment of the interfacial defect, admittance spectroscopy measurements under varying biases were conducted to investigate the defects within the reference and Cs-treated samples. Figure 2a–d illustrates the admittance spectra of the increased amount of Cs-treated samples under zero bias. In the case of ref-CIGSSe (Figure 2a), the absence of a capacitance step in the spectra suggests the absence of any discernible defects within the devices. On the other hand, from Figure 2b–d, the main capacitance step shifts to a lower frequency, indicating the presence of deeper defects as highlighted in the regions with a blue backdrop. The Arrhenius equation (see Figure S2) was utilized to quantify the defect activation energy,^25^ as summarized in Figure 2e. As the Cs incorporation increases, the dominant defects in the CIGSSe absorber become more pronounced and deeper. In the case of the Overdose_Cs-CIGSSe device, the presence of an *N2* defect, which is recognized as detrimental to the device quality, is especially notable. In addition, with the increase in Cs incorporation, there is an observed rise in the capacitance gap between high (*C*_hf_) and low frequency (*C*_lf_). The high and low-frequency capacitances were extracted at frequencies of 10^7^ Hz and 10 Hz, respectively, at 200 K. The expanded capacitance gap likely corresponds to a thicker blocking barrier width (*W*_b_) within the devices, as reported in the reference.^26^ The estimated thickness can be assumed by eq 1:
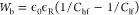
1where ϵ_0_ represents vacuum permittivity and *ϵ*_R_ denotes relative permittivity. Considering that the CIGSSe and Cs-treated CIGSSe samples may obtain different relative permittivities, our focus is solely on the Cs-treated samples. The calculated widths of the blocking barrier were 51*ϵ*_R,Cs_, 78 *ϵ*_R,Cs_, and 83 *ϵ*_R,Cs_ for Low_Cs-CIGSSe, Cs-CIGSSe, and Overdose_Cs-CIGSSe samples, respectively, where *ϵ*_R,Cs_ denotes their relative permittivity. To further assess the barrier height of the potential blocking layer for carriers traversing the p–n junction, we conducted the dark *J*–*V*–*T* curves (see Figure S3a–d) to extract the injection current barrier *E*_A_. This parameter can be considered equivalent to the conduction band offset (CBO) at the window (and buffer)/absorber interface.^26^ For the Cs-treated devices, the *E*_A_ values were determined to be 81.6, 104.8, and 107.2 meV for Low_Cs-CIGSe, Cs-CIGSSe, and Overdose_Cs-CIGSSe devices, respectively. The increase of *E*_A_ after alkali metal incorporation has also been reported in Rb and other Cs-treated studies.^26,27^ In other words, increased Cs incorporation can effectively induce the CBO, which is in line with our previous estimation of the blocking layer thickness. The barrier thickness increases, resulting in an increase in the barrier height for current injection at the p–n junction. Nevertheless, note that the alkali-induced blocking barrier *W*_b_ may not solely originate from the p–n interface but could also involve the back contact or extend into the grain boundaries throughout the bulk material. The accumulation of heavy alkali metal ions at the back contact has also been observed through PDT.^18^ Additionally, the intentional incorporation of heavy alkali metal at the backside has deteriorated the FF by increasing *R*_s_.^18,30^ In the sample of Cs-CIGSSe, we also detect a Cs signal at the backside between CIGSSe and the MoSe_2_ interface (see Figure S4). These findings imply a strong correlation between the Cs-induced barrier with the degree of Cs incorporation.

Furthermore, the bias-admittance measurements were performed as shown in Figure S2. The defect activation energies (*E*_a_) under various forward biases for both reference and Cs-treated devices were obtained, as depicted in Figure 2e. We observed that the variation of extracted *E*_a_ fluctuates with the condition of Cs incorporation. One of the extracted *E*_a_ under zero bias in Overdose_Cs-CIGSSe vanishes after adding forward bias; in addition, the Overdose_Cs-CIGSSe sample exhibits significant fluctuations in the extracted *E*_a_ values, including the *N2* defect examined at zero bias, when different forward biases are applied. In contrast, the Low_Cs-CIGSSe and Cs-CIGSSe samples consistently display identical values of *E*_a_ even though we changed different forward biases. The data indicate that defect traps in the Low_Cs-CIGSSe and Cs-CIGSSe samples are uniformly distributed within the depletion region. Both the p–n interface and the space charge region exhibit similar characteristics. However, in the case of Overdose_Cs-CIGSSe, this can exacerbate the disparity in defect characteristics between the interface and the inner depletion region.

**Figure 3:**
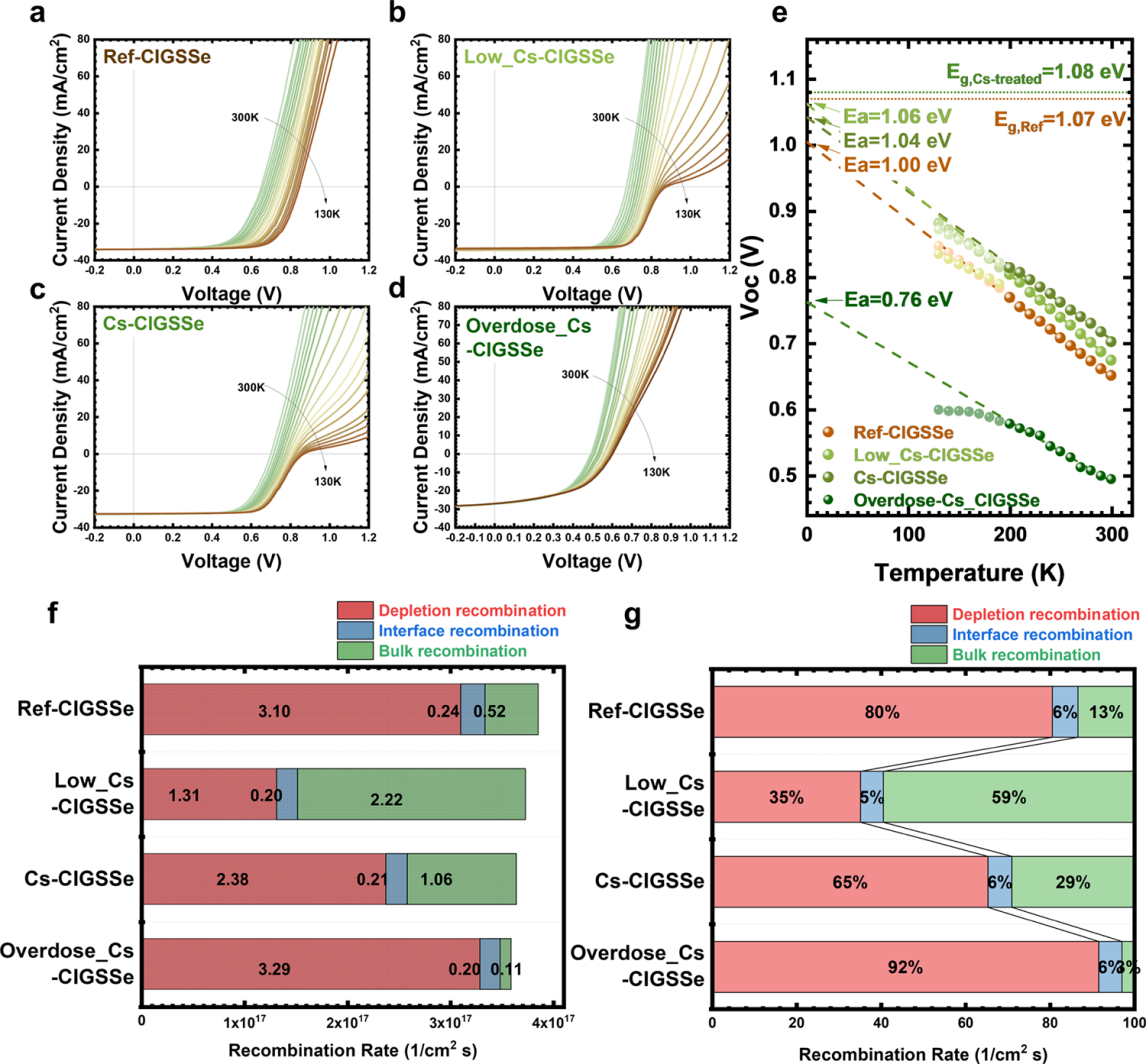
Temperature-dependent *J–V* measurement of (a) ref-CIGSSe, (b)Low_Cs-CIGSSe, (c) Cs-CIGSSe, and (d) Overdose_Cs-CIGSSe devices under illumination. (e) *V*_oc_*–T* measurement of different amounts of Cs-treated devices. (f) Recombination rates of depletion, interface, and bulk recombination derived from *Suns-V*_oc_ measurements. (g) Normalized recombination rates in percentage.

Temperature-dependent *J–V* (*J–V–T*) measurements were further carried out to investigate their recombination behaviors. In Figure 3a–d, the *J–V–T* curves under illumination display various diode responses with temperature. Although the devices with Cs incorporation mostly illustrate rollover under low-temperature regions, merely Overdose_Cs-CIGSSe shows relatively minor current blocking at a high sweep forward voltage. Also, when we extracted the activation energy (*E*_a_) from the plot of *V*_OC_ vs temperature by extrapolation toward 0 K, as shown in Figure 3e. Overdose_Cs-CIGSSe shows the smallest value (*E*_a, Overdose_Cs-CIGSSe_: 0.76 eV) compared with those of the other devices (*E*_a, ref-CIGSSe_: 1.00 eV; *E*_a, Low_Cs-CIGSSe_: 1.06 eV; *E*_a,Cs-CIGSSe_: 1.04 eV). The extracted *E*_a_ can be used to evaluate the dominated recombination by comparing it with an energy bandgap (*E*_g_).^28^ Obviously, Overdose_Cs-CIGSSe is notably influenced and predominantly marked by interface issues, given its considerable deviation from *E*_g_; in contrast, Low_Cs-CIGSSe and Cs-CIGSSe align closely with *E*_g_, indicating a predominance of bulk or depletion recombination behavior. Illumination-dependent Suns-*V*_OC_ was further used to calculate their recombination rate within the thin film device.^29^ As shown in Figures 3f and S5, the calculated recombination rates of the interface are identical across all four samples. However, the rates of depletion and bulk recombination represent substantial differences. Therefore, besides comparing their recombination rates, the relative ratio of recombination can effectively distinguish their contributions, as depicted in Figure 3g. Low_Cs-CIGSSe shows the smallest ratio in interface recombination and the highest ratio in bulk recombination. Among the same ratios in interface recombination of ref-CIGSSe, Cs-CIGSSe, and Overdose_Cs-CIGSSe, Cs-CIGSSe obtains the highest ratio of bulk recombination. The trends observed in the ratios of interface and bulk recombination align with those of the final PCEs. That is, PCEs exhibit a strong dependence on recombination behaviors, indicating that the best performance may not necessarily be associated with the highest *V*_*OC*_ but rather the best FF.

As previously discussed, samples with Cs incorporation exhibit the presence of a potential barrier for carrier injection blocking. Cs incorporation induces a thicker blocking layer, higher *E*_A_, and deeper *E*_a_, which directly impacts the recombination behavior within the device.^26^ The elevation in the injection barrier with the augmentation in the quantity of alkali-PDTs, such as KF-PDT and RbF-PDT, have also been documented in previous reports.^26,27,30^ Furthermore, this change often coincides with significant surface modifications, encompassing alterations in morphology, grain boundary properties, and even the formation of new compounds. Thus, we further conducted scanning electron microscopy (SEM), Kelvin probe force microscopy (KPFM), X-ray photoemission spectroscopy (XPS)-related analysis, and transmission electron microscopy (TEM) characterizations for the following examinations.

### Unveiling Surfaces and Grain Boundaries in Cs-Incorporated CIGSSe

**Figure 4:**
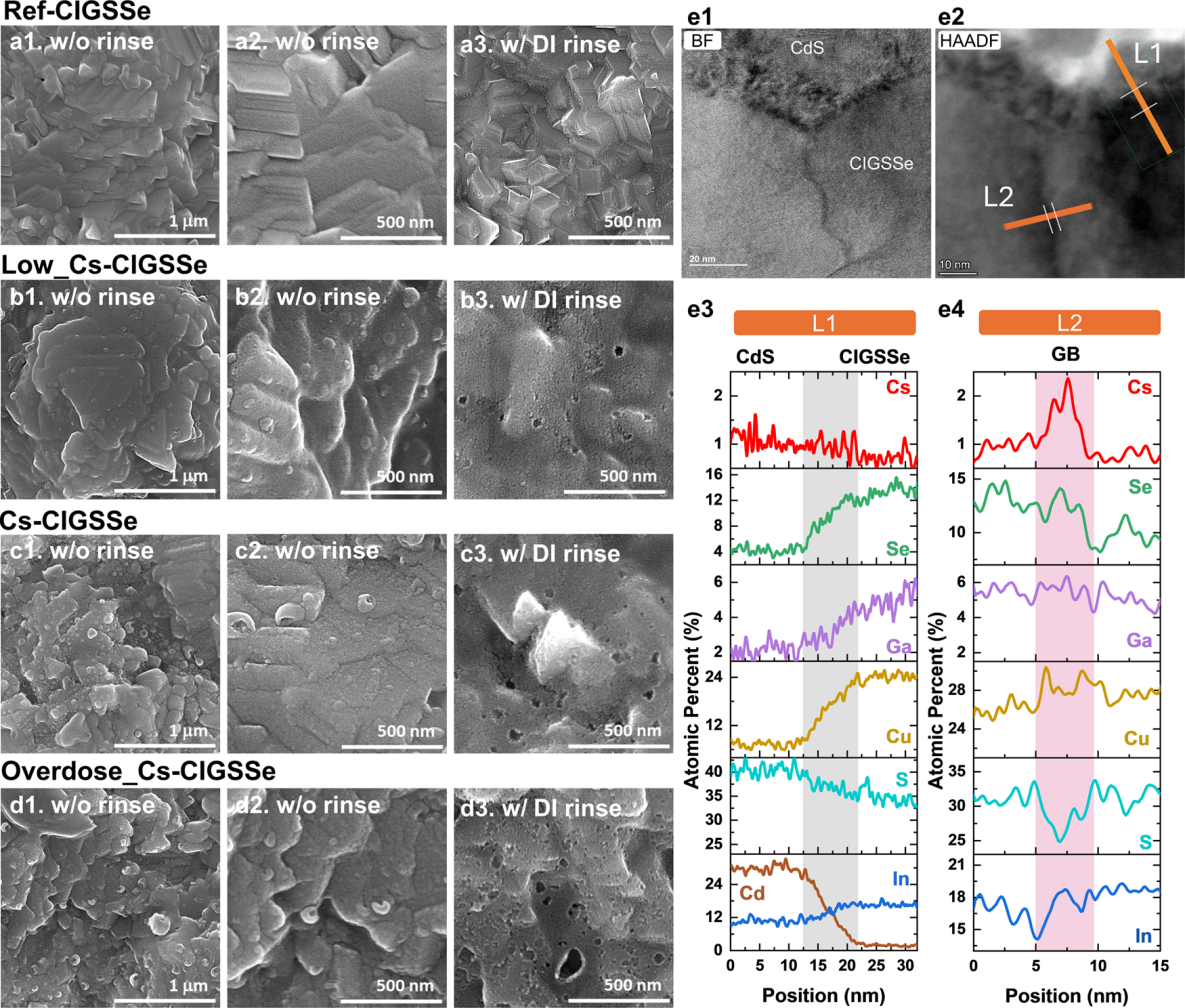
SEM top view image of different amounts of Cs incorporation on CIGSSe absorber without and with DI water rinse. (a1)–(a3) ref-CIGSSe, (b1)–(b3) Low_Cs-CIGSSe, (c1)–(c3) Cs-CIGSSe, and (d1)–(d3) Overdose_Cs-CIGSSe samples. (e1) TEM bright field (BF) image and (e2) high angle annual dark field (HAADF) image at the CdS/CIGSSe interface of the Cs-CIGSSe device. (e3) EDS line scan through CdS/CIGSSe as L1 indicated in (e2). (e4) EDS line scan through the grain and grain boundary near the front interface as L2 indicated in (e2).

Figure 4a shows the surface image of ref-CIGSSe; Figure 4b–d corresponds to Low_Cs-CIGSSe, Cs-CIGSSe, and Overdose_Cs-CIGSSe (without and with DI water rinse), respectively. Even at lower magnification (see Figure 4a1–d1), we already can observe that Cs incorporation has a substantial impact on surface morphology. The well-defined facet grains (Figure 4a2) observed in the reference have transformed into grains with rounded corners, indicating the possible formation of a new surface layer. In addition, we observed that residual small islands are randomly distributed (see Figure 4b2–d2). After DI water rinsing, the presence of small pores is present across the entire surface under high magnification (see Figure 4b3–d3), with the pore size and numbers increasing proportionally to the degree of Cs incorporation. The observation suggests that the pore size may be linked to the amount of residual CsF remaining after PDT.

The formation of nano pinholes after alkali-fluoride PDT is well-known in the cases of KF-PDT^31^ and RbF-PDT.^32^ In the case of CsF-PDT, some studies have reported the presence of nanopores and pinholes after Cs treatment,^23^ while others have found the absence of significant surface nanopores following moderate CsF-PDT.^14,33^ In the latter case, Cs compounds segregate at grain boundaries, a phenomenon that may be attributed to the relatively large ionic radius of Cs metal ions, limiting their diffusion into the grain interior or their presence on the grain facet surface.^14,33^ However, in our S-based CIGSSe absorbers, even with lower levels of Cs incorporation, noticeable changes, such as the formation of nanopores, occur across the entire surface (refer to Figure 4b). It is important to note that although the Cs incorporation method employed here is the same as the standard PDT method used in three-stage evaporation processes,^14^ the CIGSSe grains with S-rich surface exhibit obvious surface roughening. We suggest that the S-rich surface may vary the reaction affinity between Cs and the chalcopyrite surface, resulting in such a comprehensive surface change. High-resolution transmission electron microscopy (HR-TEM) coupled with energy-dispersive spectroscopy (EDS) was further employed to characterize the surface composition through line scans, as shown in Figure 4e1–e3. The bright field (BF) image and high angle annual dark field (HAADF) image both depict a nearly 10 nm transition layer mixed with CdS and CIGSSe (see line L1 in Figure 4e3). In this region, we can observe a clear signal decline in Cu and Ga, while In and VI-A groups (S+Se) show relatively constant profiles and almost no Cs are recognized. Our data indicate that the surface roughening may result from the Cu and Ga depletion, further inducing the S-based ordered-vacancy compounds (OVC) layer. The size and number of nanopores after DI water rinsing are likely influenced by the removal of varying amounts of residual CsF.

**Figure 5:**
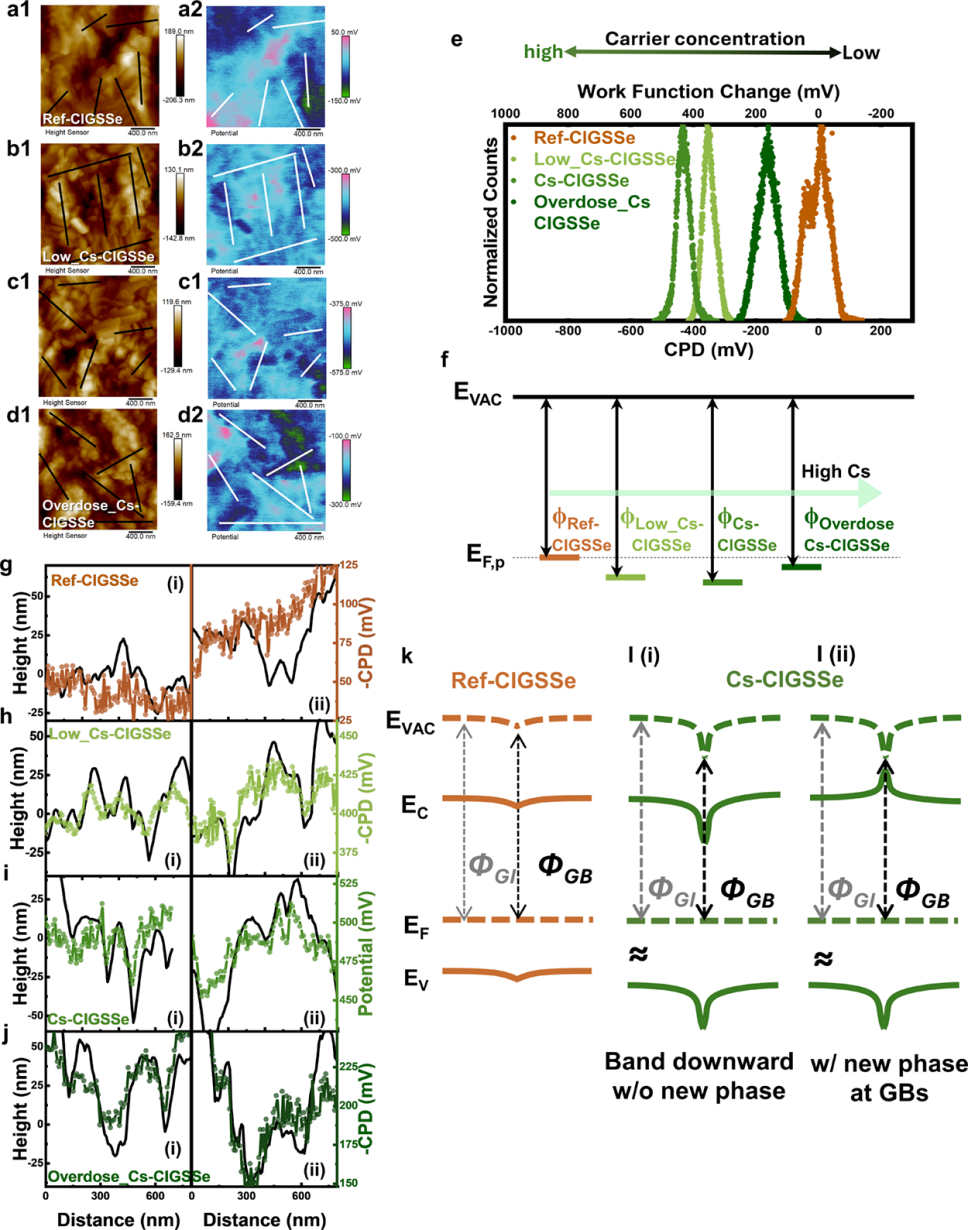
KPFM measurement of (a1–d1) topography and (a2–d2) contact potential difference (CPD). (a1)–(a2) ref-CIGSSe, (b1)–(b2) Low_Cs-CIGSSe, (c1)–(c2) Cs-CIGSSe, and (d1)–(d2) Overdose_Cs-CIGSSe absorber samples. (e) Normalized *CPD* distribution and (f) schematic band diagram model of Reference and Cs-treated samples. Selected height profiles and potential profiles across grain boundaries of (g) ref-CIGSSe, (h) Low_Cs-CIGSSe, (i) Cs-CIGSSe, and (j) Overdose_Cs-CIGSSe absorber samples. Energy band diagrams at the grain boundary of (k) ref-CIGSSe and (l) Cs-CIGSSe samples. (i) without new phase and (ii) without new phase.

As Cs incorporation impacted the entire surface, KPFM measurements were further conducted to gain a deeper understanding of the properties of the grain boundaries. Figure 5a1–d1 shows the topography of ref-CIGSSe, Low_Cs-CIGSSe, Cs-CIGSSe, and Overdose_Cs-CIGSSe, respectively. Figure 5a2–d2 corresponds to their contact potential difference (CPD) mappings. Figure 5e illustrates the summarized CPD histogram. The change in work function can be calculated using the equation CPD = (φ_tip_ – φ_sample_)/*e*, where φ_tip_ represents the work function of the tip and φ_sample_ denotes the work function of the sample, with *e* being the elementary charge. The relative position of the work function therefore can be depicted in a scheme such as Figure 5f. The work function initially increases with the addition of a low level of Cs to CIGSSe, reaching its highest in the median dose of Cs-CIGSSe; finally, a slight decline is observed when an excess amount of Cs is incorporated in Overdose_Cs-CIGSSe.

Fluctuations in the work function may signify changes in the carrier concentration following Cs incorporation. A higher work function in p-type semiconductors may indicate an increased carrier concentration. Alkali metal ion incorporation is usually accompanied by Cu depletion and substantial *V*_Cu_ creation. These Cs-induced *V*_Cu_ defects can effectively contribute to the p-type carrier concentration. A similar result—an elevated work function and increased carrier concentration—was also observed in KF-PDT in CIGS solar cells.^34^ In the case of excess Cs incorporation leading to a decline in carrier concentrations, this phenomenon may originate from ion-exchange mechanisms or variations in surface compositions, as reported in Cs and other alkali metal incorporation in CIGS solar cells.^35^ Although the work function change in our case aligns with the tendency observed in *N*_CV_, TEM analysis of the grain surface composition and KPFM surface characteristics suggests that the change is more closely linked to alterations in the S-based OVC layer. This correlation implies that the alkali-induced OVC layer may play a significant role in determining carrier concentration, rather than solely the presence of alkali metal ions.

Besides the overall surface contact potential, the CPD across grains and grain boundaries also shows discrepancies among the samples without and with Cs incorporation. Figure 5g**–**j shows extracted potential profiles corresponding to ref-CIGSSe, Low_Cs-CIGSSe, Cs-CIGSSe, and Overdose_Cs-CIGSSe, respectively. Six heights and their corresponding potential profiles across the grain and grain boundary are extracted from the potential and height mapping as shown in Figure S6, and selected two potentials are illustrated in Figure 5g–j and denoted as (i) and (ii). Reduced-height regions are considered grain boundaries, while increased-height regions are considered grain interiors. It is noted that the right *Y*-axis is transferred minus CPD (−CPD) to represent the work function change. In the reference sample (refer to Figure 5g), the potential profiles deviate from the height profiles. Conversely, as Cs are incorporated, the potential profile tends to gradually align with the height profile (see Figure 5h–j), indicating a more pronounced decrease in the work function at grain boundaries. This suggests that the band bending at the grain boundaries tends to bend downward, resulting in hole barriers.^34^ The upward and downward band bendings at the grain boundaries can act as electron barriers and hole barriers, respectively. However, in high-performance solar cells, both upward and downward band bending at grain boundaries from KPFM results both have been observed.^36^ The result reflects that the work function change may not accurately represent the real band bending if there are new phases or compounds at grain boundaries that differ from those on the grain surface. The key to improving performance may lie in how alkali modulation induces effective grain boundary passivation to reduce defect density.

It has been reported that both light and heavy alkali metal ions tend to aggregate along the grain boundaries.^14,16−18^ Alkali metal ions located at grain boundaries tend to reduce charged defect density, possibly due to the presence of secondary phases.^19^ The emergence of a new phase with a large bandgap may serve as both electron and hole barriers, effectively diminishing carrier recombination. Therefore, in our study grain boundaries may undergo passivation through Cs incorporation via the formation of a new phase. Indeed, in the L2 line scan shown in Figure 4e4, Cs presence at the grain boundary near the front interface was noted, along with relatively constant signals of Cu, In, Ga, and Se. Remarkably, the composition distribution is different from the grain surface; there is a noticeable decrease in S at the grain boundary. This observation suggests a preference for Se over S at grain boundaries, resulting in a Se mixed low-S phase compared to that on the grain surface. We hypothesize that this may stem from Cs–In–Se-related compounds having a lower formation energy than Cs–In–S-related phases.^37^ As Cs segregates at the grain boundaries, it may tend to react with Se and deplete S. In fact, a simulated value of 2.66 eV for the CsInSe_2_ compound,^38^ which represents the largest theoretical value reported for a CIGS-based absorber with alkali treatment, suggests its potential presence at grain boundaries. If this compound indeed exists at grain boundaries, it could elevate the conduction band minimum (CBM) and lower the valence band maximum (VBM). The decrease in the work function at grain boundaries may further indicate that the new phase at grain boundaries has a smaller electron affinity if the CBM is elevated due to the new phase.

**Figure 6:**
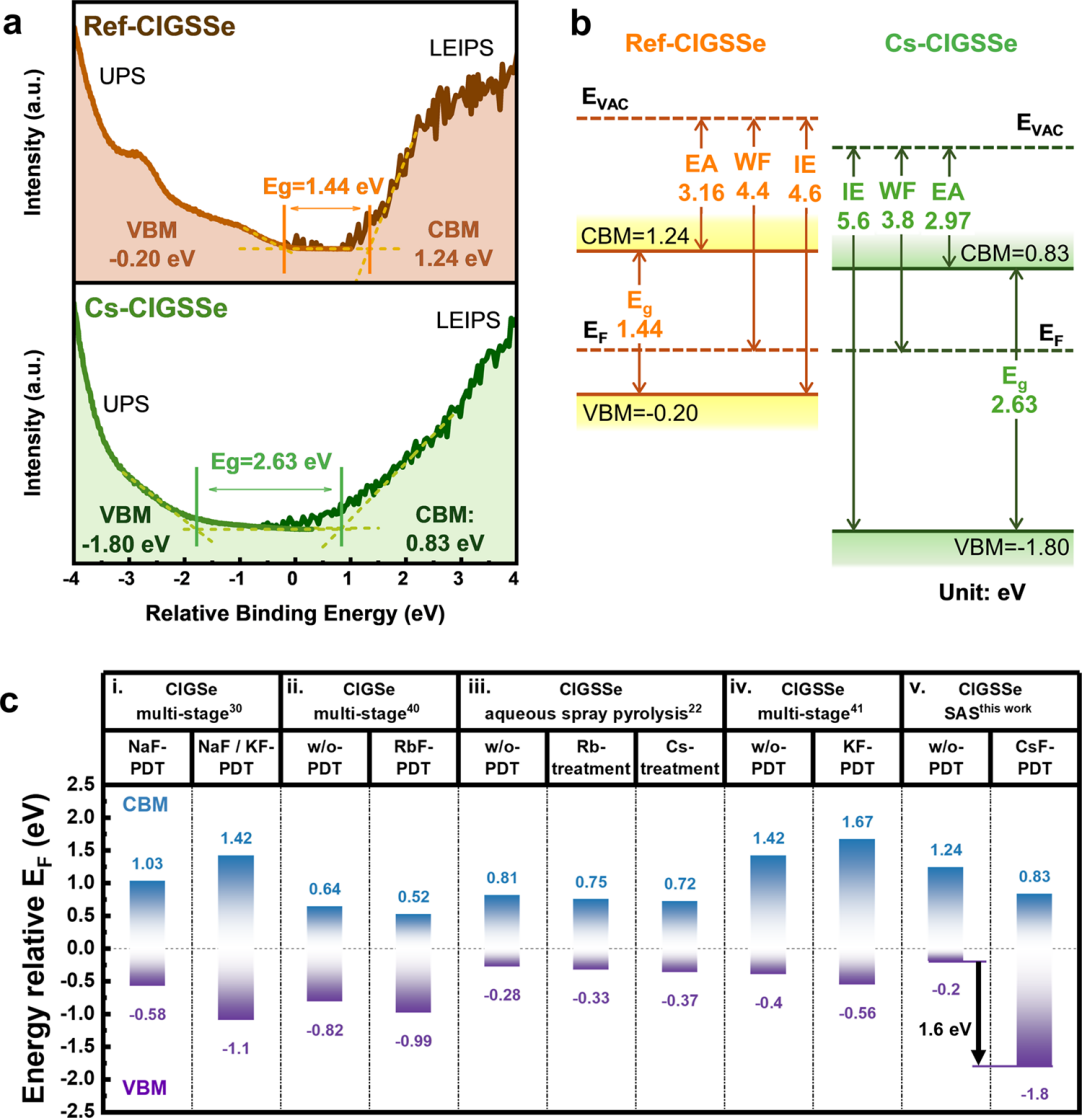
UPS and LEIPS measurement for front interface bandgap of (a) ref-CIGSSe and Cs-CIGSSe samples. (b) Schematic diagram of the energy band diagram of ref-CIGSSe and Cs-CIGSSe samples. EA, WF, IE, and *E*_g_ denote electron affinity, work function, ionization energy, and bandgap, respectively. (c) Measured CBM and VBM of alkali treatment on CIGS-based absorbers. (i) VBM and CBM obtained by UPS and inverse photoelectron spectroscopy (IPES). Reproduced with permission from ref (30). Copyright 2015 American Chemical Society. (ii) UPS and IPES. Reproduced with permission from ref (40). Copyright 2017 American Chemical Society. (iii) UPS and Au calibration. Reproduced with permission from ref (22). Copyright 2023 American Chemical Society. (iv) UPS and IPES. Reproduced with permission from ref (41). Copyright 2017 AIP Publishing. (v) UPS and LEIPS ^this work^.

Given the observed changes in the KPFM and TEM-EDS results, there is a notable interest in determining the positions of the CBM and VBM after Cs incorporation. To examine the aforementioned changes in the energy band, ultraviolet photoelectron spectroscopy (UPS) and low-energy inverse photoemission spectroscopy (LEIPS) were employed to verify and quantify the band offset on the reference sample (ref-CIGSSe) as well as the sample with Cs incorporation (Cs-CIGSSe). UPS and LEIPS spectra are shown in Figure 6a. The details of reconstructing the energy band diagram are illustrated in Figure S7. According to Figure 6b, with Cs incorporation, the position of the CBM shifted downward by 0.41 eV with respect to the Fermi level; in contrast, the position of the VBM shows a significant downward shift of 1.6 eV in Cs-CIGSSe sample. Therefore, the bandgap of the sample rises from 1.44 eV for ref-CIGSSe to 2.63 eV for the Cs-CIGSSe sample. For the S-containing Cu(In, Ga)(S, Se)_2_ absorber, the surface bandgap of ref-CIGSSe was calculated to be 1.50 eV using an empirical formula (eq S6).^39^ The calculation was performed in conjunction with the atomic ratios obtained from the XPS measurement, as illustrated in Figure S8 and Table S3. The energy bandgap constructed in our study closely aligns with the calculated value for the reference sample, affirming the credibility of our energy band construction. On the other hand, the observed 2.63 eV in Cs-CIGSSe indicates an enlarged energy bandgap exceeding 1 eV, which is significantly larger than the bandgap of the current Cs-incorporated or any other alkali-treated CIGS-based solar cells.^22,30,40,41^ Furthermore, it is reasonably close to the theoretical value of 2.66 eV for the CsInSe_2_ compound.^38^ The binding energies of In 3d, Se 3d, and S 2p from XPS spectra also demonstrate significant shifts (see Figure S8). The In 3d_5/2_ in Cs-CIGSSe shows an increased intensity (denoted as In 3d_5/2_-1) at higher binding energy (BE); additionally, Se 3d, and S 2p display additional peaks at lower binding energies (denoted as Se 3d_3/2_-2, Se 3d_5/2_-2, S 2p_1/2_-2, S 2p_3/2_-2, Se 3p_1/2_-2, and Se 3p_3/2_-2, as shown in Figure S8). These shifts in binding energies resemble the characteristics observed in the formation of K–In–Se^42^ or Rb–In–Se.^43^ Although recent research suggests that the improvements in PCE may not be solely attributed to the formation of the alkali–In-Se_2_ phase but rather to the exchange of Cu with alkali metal in OVC,^43^ our data provides evidence of new bond formation, regardless of whether it is a stoichiometric alkali–In-Se_2_ phase or not. This all contributes to widening of the surface bandgap. In addition, combined with our TEM-EDS analysis, which reveals different compositions at grain surface and grain boundaries in Figure 4e3,e4, the observed bandgap widening may be an averaged outcome influenced by regions with and without Cs-compound. The averaged CBM downshift may mostly result from the OVC, for which Se-based OVC has been reported to decrease CBM compared to pristine CIGS; in contrast, S-based OVC and a high *E*_g_ Cs-compound may both contribute to a significant VBM downshift. That is, the S-based OVC on the grain surface and Cs-compound at grain boundaries both construct such high *E*_g_ of 2.63 eV, which has not been investigated to date.^30,40^

However, we observed a disparity in the work function change between KPFM and UPS measurements. KPFM suggests an increase in the work function after Cs incorporation, whereas the work function calculated from UPS indicates a decrease. According to their experimental environments, we propose that this discrepancy may stem from variations in the measurement background conditions. KPFM measurements predominantly reflect the material’s surface potential under nearly dark conditions, aligning more closely with results from capacitance spectroscopy, such as the aforementioned *C–V* measurements. On the contrary, UPS measurements depend on UV photons to eject electrons to determine the energy position of the Fermi level. In essence, during UPS measurements, the sample is exposed to UV radiation to gather band information. However, the UV absorption in the CIGS absorber may also generate photocarriers, potentially interfering with the determination of the work function. In fact, certain reports have noted that UPS typically shows an average lower work function compared to KPFM.^44−46^ Although debates may arise regarding the accurate value of the work function or the corresponding Fermi level during UV light exposure, the reconstructed VBM and CBM remain reliable. Most bandgap constructions on different alkali metal incorporations in CIGS-based absorbers were measured using a UPS-related method as shown in Figure 6c.^22,30,40,41^ Our work demonstrates the largest VBM downshift for state-of-the-art alkali-treated CIGS-based samples.

Combining the enlarged surface bandgap from UPS/LEIPS and the changes in work function at grain boundaries (GBs) from KPFM, diagrams for grain boundaries in ref-CIGSSe and Cs_CIGSSe are schemed in Figure 5k,l, respectively. Considering the work function changes from KPFM, ref-CIGSSe exhibits almost no specific band bending toward the grain boundary (refer to Figure 5g). Conversely, in Cs-treated CIGSSe without new phase formation, GBs may display a strengthened downward band bending due to decreased work function, as illustrated in Figure 5l(i). However, with potential new Cs-related phases observed at GBs in TEM-EDS results, both the CBM and VBM could be extended, effectively mitigating carrier accumulation at GBs, as depicted in Figure 5l(ii). The upward shift in the conduction band can further reduce electron accumulation at the GBs, while the downward shift in the valence band can effectively reduce the Shockley–Read–Hall (SRH) recombination at GBs, approaching the grain-boundary-free model.^47^ This GB structure can reduce the density of charged defects,^39^ explaining how proper Cs incorporations can effectively enhance *V*_OC_ in Cs-treated CIGSSe samples. Nevertheless, considering the investigation from the interface study, despite pronounced GB engineering, a thick blocking layer could also have adverse effects if it generates barriers at the front and even backside interfaces. Thus, while Cs incorporation may elevate *V*_OC_ through GB engineering, it could also introduce additional recombination at the interface, depletion, and even the bulk regions.^31^

The comprehensive analysis integrates considerations of the interfacial blocking layer, surface bandgap widening, and grain boundary optimization. The most successful Cs-incorporated CIGSSe device, combined with an antireflective coating, achieved an efficiency of 18.11% (see Figure S9). Notably, the best-performing cell did not necessarily attain the highest *V*_OC_, as the enhanced FF through an appropriately tuned blocking barrier width significantly contributed to and elevated the overall efficiency. While the current p–n junction may still exhibit a cliff-like conduction band offset *(E*_c_*– E*_F, CIGSSe with Cs incorporation_ = 0.86 eV; *E*_c_*– E*_F, CdS_ = 0.5 eV^48^), offering potential for further improvement in band alignment with new buffer layers, the thinner blocking layer may additionally decrease interface recombination. Moreover, our results indicate that Cs incorporation can enlarge the surface bandgap by over 1 eV, particularly shifting the VBM downward. This suggests that heavy alkali metal ions, such as Rb and Cs, own the ability to effectively engineer the VBM. Furthermore, Cs can further shift the VBM downward to nearly 1.6 eV. Such control of the VBM is crucial for designing hole transport in solar cells^49,50^ or adjusting the photoelectrode/electrolyte interface in water-splitting applications.^51^ Also, as we found the composition distribution, Cs induces S depletion at GBs, indicating the binding affinity with Se. The strong binding affinity with Se may also intensify the formation of S-based OVC. Cs repels Cu and Ga but does not stay with the S-rich grain surface during the migration to GBs. The preference for Se in Cs-treated CIGSSe absorbers offers increased opportunities for surface and grain boundary engineering, providing greater flexibility in tuning the surface bandgap. Once this elevated surface bandgap, which surpasses even that of CuGaSe_2_ (1.68 eV), is appropriately aligned with a buffer layer, the *V*_OC_ has the potential to surpass the record of 1 V.^52^ Also, the presence of n-type characteristics at the front interface facilitates the formation of a quasi-homojunction. This quasi-homojunction may improve band alignment with the subsequent buffer layer, enabling the establishment of a deeper junction. Moreover, it may even eliminate the need for an additional n-type buffer layer, thereby enhancing photon collection from low-wavelength regions. For future multiple full chalcopyrite tandem solar cells, the high bandgap modulation also provides new insights into matching the current density as the top cell. Furthermore, beyond the field of photovoltaics, this research holds potential applications in photochemical research, such as the photoelectrode/electrolyte interface in water splitting. The modulated surface bandgap can adjust to match the redox potential for better hydrogen/oxygen evolution reactions. These findings showcase its significance, even beyond the realm of photovoltaics.

## Conclusions

In this study, we thoroughly investigated the influence of Cs incorporation on the S-containing CIGSSe absorber. The best efficiency with antireflection coating approached 18.11%. The modified surface, featuring S-based OVC and Cs-containing GBs, resulted in an enlarged surface bandgap from 1.44 to 2.63 eV. Cs incorporation particularly downshifts VBM by nearly 1.6 eV, marking the most significant alteration of the VBM observed thus far. The enhancement in *V*_OC_ with Cs incorporation can be ascribed to grain boundary engineering facilitated by the formation of a Cs-compound, along with the widened surface bandgap. The top-down postdeposition treatment involving Cs incorporation affects not only the junction interface but also the backside and may extend into the grain boundaries throughout the entire bulk material. Electrical analysis revealed that proper Cs incorporation in CIGSSe led to the suppression of interface recombination. The recognition of Se preference at the Cs-containing GBs in CIGSSe provides significant insights into chalcogenide systems coexisting with both S and Se, opening avenues for advanced surface or grain boundary engineering.

## Experimental Methods

### Sample Preparation

Mo films were grown on a soda-lime-glass (SLG) substrate. A 1.5-μm CIGSSe absorber was fabricated by using a sequential process. The sputtered CuGa/In precursors were first selenized and then underwent sulfurization. The stacked precursors were heated at around 550 °C with a heating ramp rate of 20 °C/min and maintained for 30 min under Se vapor (Se pellet). The sulfurization process was further heated to 590 °C at a ramping rate of 20 °C/min and maintained for 30 min under a pressure of 1 atm using a mixture of 5% H_2_S in Ar gas. After the sequential process for the fabrication of the CIGSSe absorber, the samples were sent to an evaporator with a background pressure of approximately 3 × 10^–8^ Torr. The Cs incorporation into CIGSSe via the CsF-PDT process was conducted with K-cell temperatures set at 370, 380, and 430 °C for the Low_Cs-CIGSSe, Cs-CIGSSe, and Overdose_Cs-CIGSSe samples, respectively. Initially, the CIGSSe absorbers were heated to 325 °C, followed by Cs incorporation at varying K-cell temperatures for 5 min. Subsequently, they were annealed in a vacuum at 325 °C for 5 min and then cooled in the furnace. The ref-CIGSSe sample also underwent an annealing process without the addition of Cs. Prior to the deposition of the buffer layer, the samples underwent two rounds of rinsing with deionized (DI) water. The initial rinse involved the use of hot DI water at 65 °C. The CdS buffer layer was deposited by chemical bath deposition (CBD) using CdSO_4_ (0.015 M)-NH_3_ (2.2 M)–CH_4_N_2_S (1.5 M). Subsequently, a 200 nm Al-doped ZnO (AZO, ZnO 98%-Al_2_O_3_ 2 wt %) window layer was deposited onto the CdS layer by radio frequency magnetron sputtering. Lastly, a top Ni/Al electrode with a thickness of 50 nm and 3 μm, respectively, was deposited using an electron beam evaporator with a contact mask. For the campion device, 100 nm MgF_2_ antireflection coating was deposited by an electron beam evaporator.

### Device Characterization

The current–voltage (*J–V*) measurement, capacitance–voltage (*C–V*), and drive level capacitance profiling (DLCP) measurements were done by a Keithley 4200-SCS meter. The frequency used in the DLCP measurement is 10k Hz, which is the same as the CV measurement. For DLCP profiles, the variable perturbation AC bias (δ*V*) utilized in this study ranges approximately from 0.014 to 0.14 V, while the total biases (*V*) range from −0.6 to 0.05 V. The device performance was evaluated under an AM 1.5 solar simulator (Wacom) at 300 K with a power density of 100 mW/cm^2^. The intensity of the 1000W AAA xenon lamp is calibrated by the Si-reference cell, which is certified by ISE (Fraunhofer Institute for Solar Energy Systems), Germany. The external quantum efficiency (EQE) spectrum was obtained from the EQE (Enlitech) with a 75 W xenon lamp calibrated with standard Si and Ge photodiodes ranging from 300 to 1350 nm. The admittance, bias-admittance measurements, and *V*_OC_–*T* were conducted using the Paios system with Fluxim Characterization Suite software connected with a temperature-controlled probe stage (Linkam stage HFS600E-PB4). Suns-*V*_OC_ analysis was carried out by the flash test to monitor the illumination dependence of the open-circuit voltage (*V*_OC_) at room temperature (Sinton Instruments WCT-120). Scanning electron microscopy (SEM) images were carried out under an accelerating voltage of 15 kV (Hitachi 8010). The Kelvin probe force microscopy (KPFM) studies were performed on CIGSSe samples by using amplitude-modulation mode with Bruker Dimension Icon AFM equipped with surface potential microscopy. Ultraviolet photoelectron spectroscopy (UPS) and low-energy inverse photoemission spectroscopy (LEIPS) with 4.77 eV band-pass filter energy as well as X-ray photoelectron spectroscopy (XPS) were investigated by electron spectroscopy for chemical analysis (ESCA), ULVAC-PHI PHI 5000 Versaprobe III. The depth profile of the device was examined by time-of-flight secondary ion mass spectroscopy (ToF-SIMS, ION-TOF, and TOF-SIMS V) with O_2_^+^ for sputtering and Bi^+^ for analysis. Transmission electron microscopy image and energy-dispersive X-ray spectroscopy (TEM-EDS) were carried out under an accelerating voltage of 200 kV (FEI Talos F200X, JEOL JEM-F200).

## Supporting Information Available

Additional information on the characterization of the reference and Cs-treated devices: DLCP measurement, bias-admittance measurement, low-temperature *J–V* characteristic measurement under a dark environment, TEM and SIMS analysis of Cs-treated devices, Suns*-V*_oc_ measurement, KPFM analysis, UPS and LEIPS measurement, XPS measurement, and *J–V* and EQE characteristic curves of the champion cell in this study (PDF)
