# Supplementary material for: Cesium Modulation in Cu(In, Ga)(S, Se)2 Solar Cells: Comprehensive Analysis on Interface, Surface, and Grain Boundary
Source: ACS Appl Mater Interfaces. 2024 Jun 12;16(25):32220–31. doi: 10.1021/acsami.4c03680 (PMC11212019; doi:10.1021/acsami.4c03680)
Supplement: Supplementary file 1 — am4c03680_si_001.pdf [file am4c03680_si_001.pdf]

Supporting Information

Cesium Modulation in $Cu(In, Ga)(S, Se)_2$ Solar Cells: Comprehensive Analysis on Interface, Surface, and Grain Boundary

*Yung-Hsuan Chen, Rui-Tung Kuo, Wei-Chih Lin, Chien-Yu Lai, Tzu-Ying Lin**

Department of Materials Science and Engineering, National Tsing Hua University, 101, Sec. 2, Kuang-Fu Road, Hsinchu 300044, Taiwan R. O. C.

*Corresponding author: Tzu-Ying Lin: tzuying.lin@mx.nthu.edu.tw

The drive-level capacitance profiling (*DLCP*) measurement is renowned for its diminished sensitivity to interface states. To avoid measuring interface states, both AC and DC bias are employed in the measurement. Additionally, to maintain the Fermi energy constant, the total bias should remain constant (i.e., V=constant= $V_{AC}+V_{DC}$). As the change in charge (δQ) and the perturbation in AC bias (δV) occur, C_0 and C_1 in **Equation S1** can be deduced.

The frequency used in the *DLCP* measurement is 10k Hz, which is the same as the *CV* measurement. The raw data of *DLCP* measurements are illustrated in **Figure S1.** The variable perturbation AC bias (δV) utilized in this study ranges approximately from 0.014 to 0.14 V, while the total biases (V) range from -0.6 V to 0.05 V. The extracted C_0 and C_1 from **Equation S1** are also shown in **Table S1**. Utilizing C_0 and C_1, the carrier concentration obtained from *DLCP* (N_{DLCP}) can be deduced from **Equation S2**, where q represents the elementary charge of an electron; A denotes the area of the device; and ε signifies the permittivity, with ε_R=13.6 utilized in the calculation. In addition, the profiling distance to the junction interface can refer to **Equation S (3)**, where $\langle x \rangle$ is the profiling distance.

$$\frac{\delta Q}{\delta V} = C_0 + C_1 \delta V + C_2 (\delta V)^2 + \cdots \qquad \textbf{Equation S 1}$$

$$N_{DL} \cong -\frac{C_0^3}{2q\varepsilon A^2 C_1} \qquad \textbf{Equation S 2}$$

$$\langle x \rangle = \frac{\varepsilon A}{C_0} \qquad \textbf{Equation S 3}$$

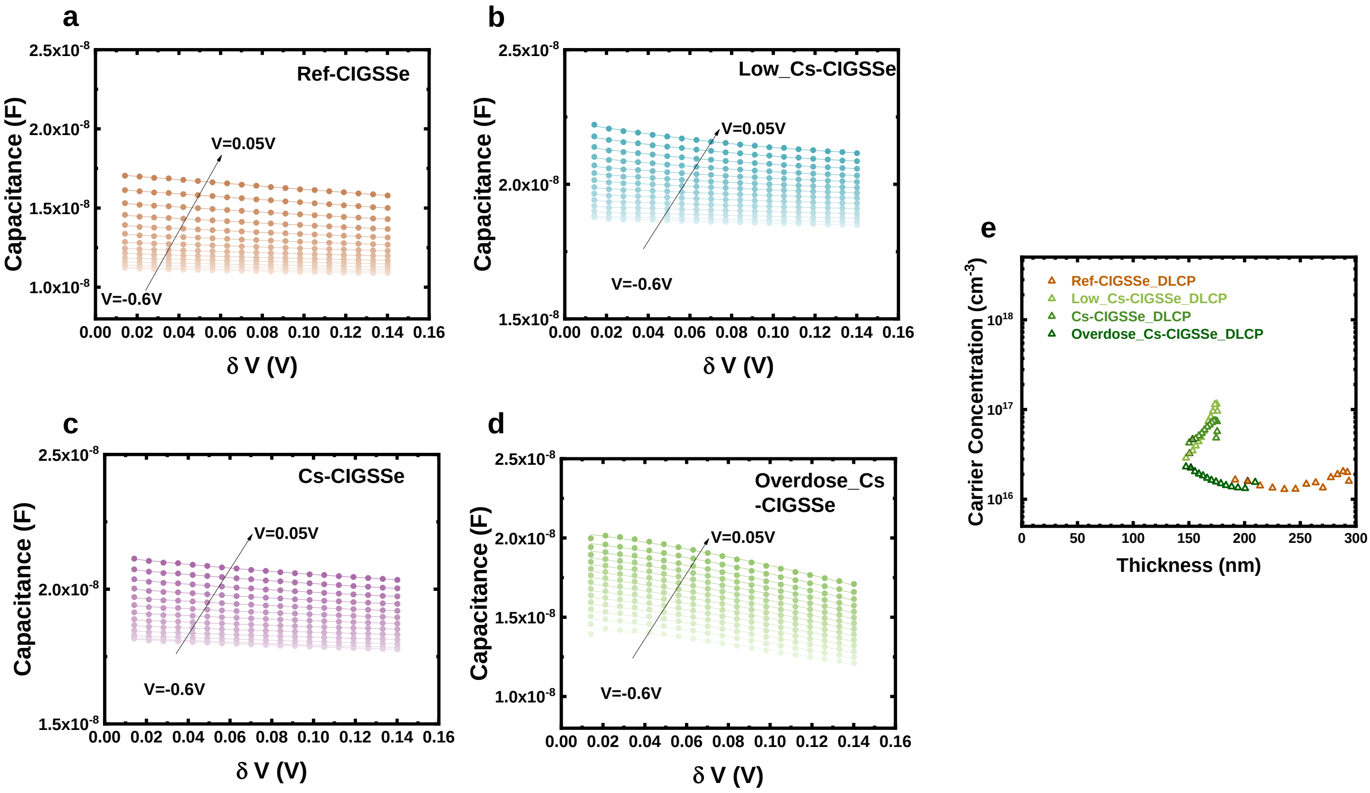

Figure S 1. Variation of junction capacitance in (a) Ref-CIGSSe, (b) Low_Cs-CIGSSe, (c) Cs-CIGSSe, and (d) Overdose_Cs-CIGSSe under varying AC bias (δV) and DC bias, measured at a frequency of 10 kHz.(e) *DLCP* characteristics curves of devices without and with CsF-PDT treatment. The black edge of the triangles indicates the measurement under zero bias voltage.

Table S 1. The C_0, C_1, *and* C_2 values under different biases were extracted from Ref-CIGSSe, Low_Cs-CIGSSe, Cs-CIGSSe, and Overdose_Cs-CIGSSe as indicated in **Figure S1** using **Equation S1.**

	Ref-CIGSSe			Low_Cs-CIGSSe			Cs-CIGSSe			Overdose_Cs-CIGSSe		
V (V)	C_0	C_1	C_2	C_0	C_1	C_2	C_0	C_1	C_2	C_0	C_1	C_2
-0.6	1.1E-08	-3.1E-09	3.6E-09	1.9E-08	-2.4E-09	2.6E-09	1.8E-08	-4.7E-09	7.4E-09	1.4E-08	-8.0E-09	-6.5E-08
-0.55	1.1E-08	-2.5E-09	1.2E-09	1.9E-08	-2.0E-09	1.5E-09	1.8E-08	-3.9E-09	6.3E-09	1.5E-08	-1.1E-08	-5.9E-08
-0.5	1.1E-08	-2.5E-09	9.6E-10	1.9E-08	-2.1E-09	1.6E-09	1.8E-08	-3.0E-09	3.4E-09	1.6E-08	-1.2E-08	-6.0E-08
-0.45	1.2E-08	-2.9E-09	1.8E-09	1.9E-08	-2.6E-09	3.3E-09	1.8E-08	-3.0E-09	3.9E-09	1.6E-08	-1.2E-08	-6.0E-08
-0.4	1.2E-08	-3.3E-09	2.6E-09	1.9E-08	-3.1E-09	4.8E-09	1.8E-08	-3.1E-09	4.2E-09	1.6E-08	-1.3E-08	-6.0E-08
-0.35	1.2E-08	-4.7E-09	7.6E-09	2.0E-08	-3.6E-09	6.2E-09	1.9E-08	-3.4E-09	5.1E-09	1.7E-08	-1.3E-08	-6.1E-08
-0.3	1.2E-08	-4.4E-09	4.6E-09	2.0E-08	-4.3E-09	8.4E-09	1.9E-08	-3.7E-09	6.2E-09	1.7E-08	-1.4E-08	-6.1E-08
-0.25	1.3E-08	-5.1E-09	4.7E-09	2.0E-08	-5.0E-09	9.6E-09	1.9E-08	-4.1E-09	6.6E-09	1.8E-08	-1.4E-08	-6.2E-08
-0.2	1.3E-08	-6.5E-09	8.8E-09	2.0E-08	-6.1E-09	1.3E-08	1.9E-08	-4.6E-09	7.8E-09	1.8E-08	-1.4E-08	-6.3E-08
-0.15	1.4E-08	-7.4E-09	9.6E-09	2.1E-08	-7.0E-09	1.7E-08	2.0E-08	-5.2E-09	9.6E-09	1.9E-08	-1.4E-08	-6.4E-08
-0.1	1.5E-08	-8.1E-09	8.3E-09	2.1E-08	-8.3E-09	2.0E-08	2.0E-08	-5.9E-09	1.0E-08	1.9E-08	-1.5E-08	-6.4E-08
-0.05	1.5E-08	-9.0E-09	7.0E-09	2.1E-08	-9.9E-09	2.5E-08	2.0E-08	-6.7E-09	1.2E-08	1.9E-08	-1.5E-08	-6.4E-08
0	1.6E-08	-9.4E-09	2.7E-09	2.2E-08	-1.1E-08	2.8E-08	2.1E-08	-7.2E-09	1.2E-08	2.0E-08	-1.5E-08	-6.8E-08
0.05	1.7E-08	-1.1E-08	4.1E-09	2.2E-08	-1.3E-08	3.6E-08	2.1E-08	-8.3E-09	1.5E-08	2.0E-08	-1.5E-08	-6.6E-08

The variation of carrier concentration following Cs incorporation may lead to the change of V_{oc}. As shown in **Equation S 4**, V_{oc} and doping density (N_A) are correlated, where Δn and n_i indicate the excess and intrinsic carrier concentration, respectively. Therefore, the elevated carrier concentration following increased Cs incorporation may lead to the enhancement of V_{oc};. Although N_{CV} and N_{DL} present a similar trend as Cs incorporation increases, we notice that the difference value of $N_{CV}-N_{DLCP}$ rises as Cs incorporation increases; also, the ΔV_{OC} (see **Equation S 5** and **Table S 2**) demonstrates a stronger dependence on N_{DL} than N_{CV}.

$$V_{OC}=\frac{kT}{q}\ln[\frac{(N_A+\Delta n)\Delta n}{n_i^2}] \qquad \textbf{Equation S 4}$$

$$\Delta V_{OC}=\frac{kT}{q}\ln(\frac{N_{A,\ CsF\text{-}PDT}}{N_{A,\ Ref}}) \qquad \textbf{Equation S 5}$$

Table S 2. Correlation summary between carrier concentration, as obtained from CV and DLCP measurements, and the corresponding increase in V_{OC} following Cs incorporation in CIGSSe.

	N_{CV} (cm^{-3})	N_{DL} (cm^{-3})	$\Delta V_{OC, CV}$ (V)	$\Delta V_{OC, DLCP}$ (V)	$\Delta V_{OC, EXP}$ (V)
Ref-CIGSSe	2.05E+16	1.58E+16	-	-	-
Low_Cs-CIGSSe	7.78E+16	3.23E+16	0.035	0.018	0.019
Cs-CIGSSe	9.20E+16	4.62E+16	0.039	0.028	0.033
Overdose_Cs-CIGSSe	4.23E+16	2.23E+16	0.019	0.009	-0.140

An increase in the N_{CV}-N_{DL} values at zero bias indicates an increment in the number of interface traps present at the CIGSSe/CdS *p*-*n* junction. Our data suggests that the Cs incorporation may lead to an increase in interfacial defects with a higher level of Cs incorporation. This elucidates that the performance degradation on Overdose_Cs-CIGSSe is not solely due to the lower free carrier concentration but also due to the emergence of severe interfacial defects at the junction.

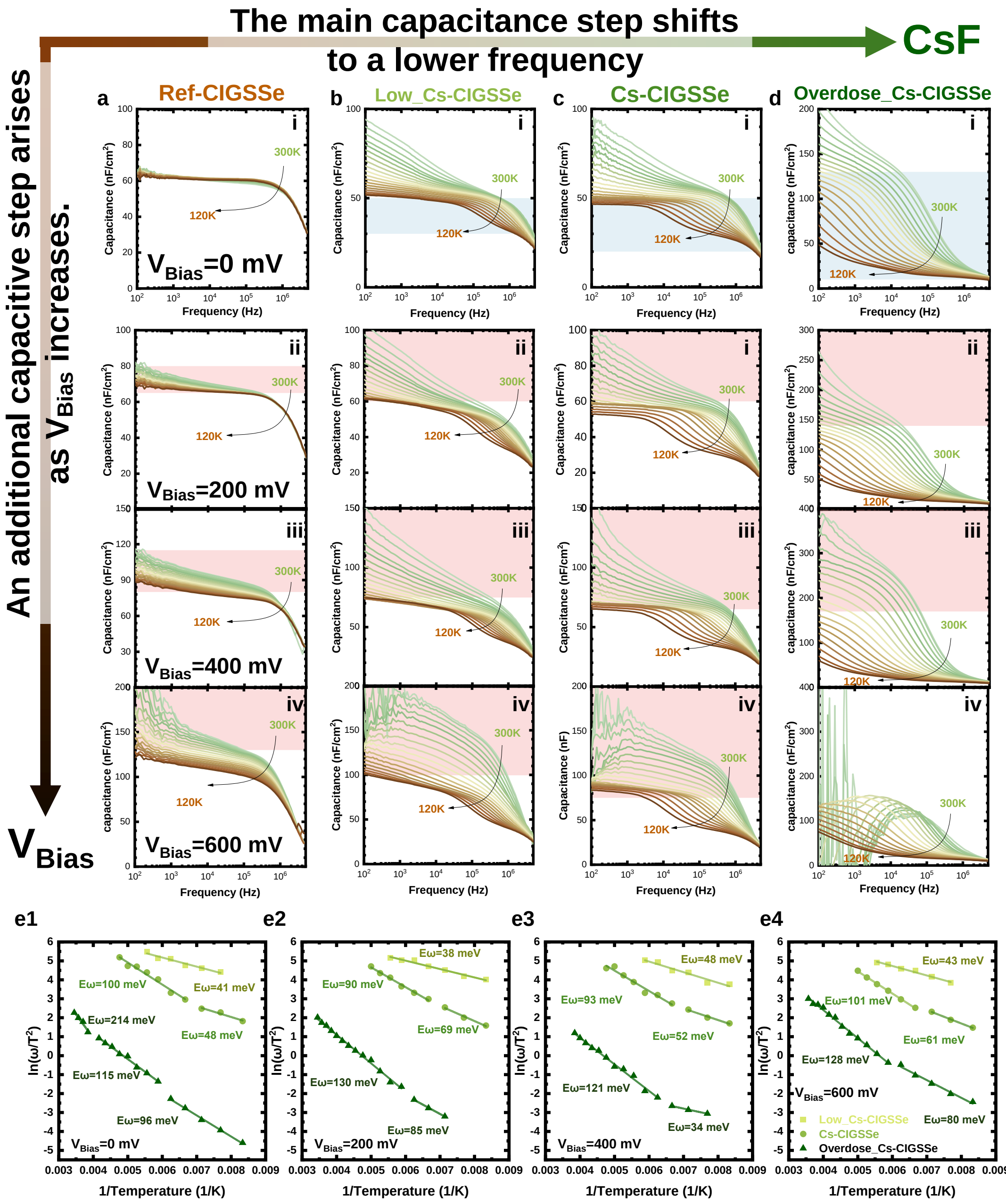

Figure S 2. Temperature-dependent bias admittance Capacitance-frequency spectra of the samples with different amounts of Cs-PDT. (a) Ref-CIGSSe, (b) Low_Cs-CIGSSe, (c) Cs-CIGSSe, and (d) Overdose_Cs-CIGSSe devices with applied bias ranging from (i) 0, (ii) 200, (iii) 400, and (iv) 600 meV, respectively. Arrhenius plots of Ref-CIGSSe, Low_Cs-CIGSSe, Cs-CIGSSee and Overdose_Cs-CIGSSe devices under (e1) 0, (e2) 200 meV, (e3) 400 meV, (d) 600meV forward bias.

The bias admittance measurements of the sample with and without Cs-treated were performed as shown in **Figure S1**. Comparing the pink backdrop under 200, 400, and 600 mV in Ref-CIGSSe, Low_Cs-CIGSSe, Cs-CIGSSe, and Overdose_Cs-CIGSSe, an additional capacitance step appears more pronounced upon raising the applied forward bias. The addition of forward bias can suppress the depletion width and alter the position of the Fermi level. Therefore, various defects in the device can be examined at different Fermi level positions, including reaching the *p*-*n* interface. For the admittance under zero applied

voltage, the defect states located near the p-n junction cannot be probed by merely AC voltage. Whereas, when applying forward bias, the potential across the *p-n* junction decreases, and the defect states located in the near interface can be probed by the Fermi level of p-CIGS under the one-side approximation[29]. As a result, the emergence of an extra capacitance step, observed as the applied forward voltage increases, is likely attributed to the presence of additional defect states near the *p-n* interface. However, if the forward bias is higher than the device's V_{OC}, the additional step may disappear at the high-temperature region due to the pronounced diffusion capacitance (see **Figure S1 (d)**). Thus, an excessive forward bias could bring considerable diffusion capacitance and destroy the capacitance-frequency profile.

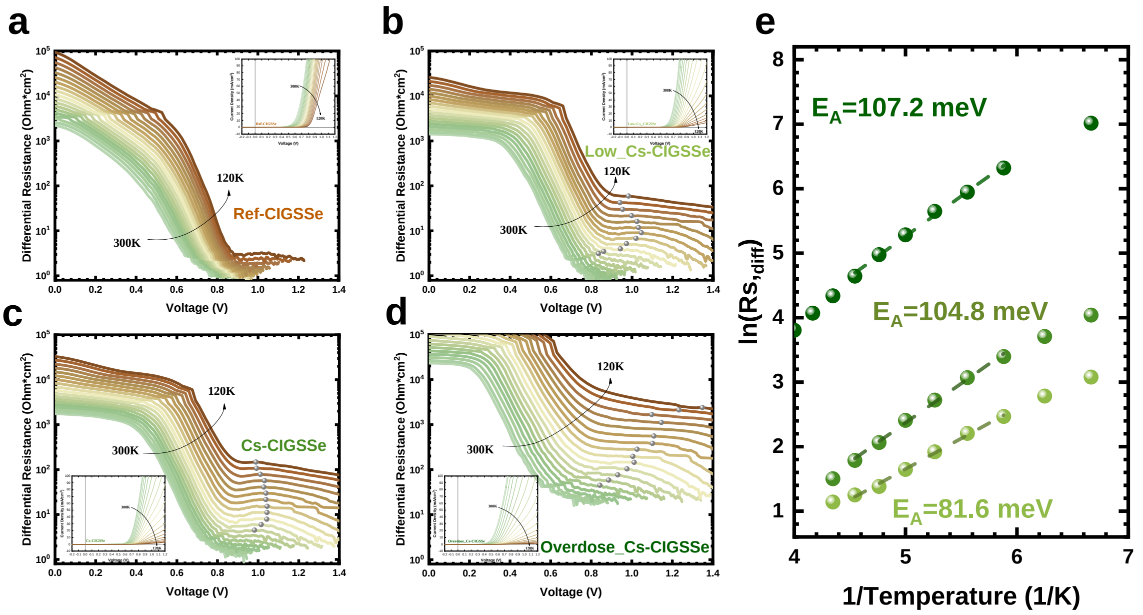

Figure S 3. Temperature-dependent *J*-*V* measurement of under dark conditions and plot as $R_{s,diff}$ -Voltage plot. (a) Ref-CIGSSe, (b)Low_Cs-CIGSSe, (c) Cs-CIGSSe, and (d) Overdose_Cs-CIGSSe devices. (e) The local maximum of $R_{s,diff}$-Voltage plot is extracted and plotted in Arrhenius plot of ln($R_{s,diff}$)-1/T plot.

The differential resistance ($R_{s,diff}$) is described as $R_{s,diff} = dV/dJ$, and the local maximum values extracted for each temperature were charted in an Arrhenius diagram. This allows us to determine the activation energy of the injection current barrier (E_A). The E_A exhibited an increase as the extent of Cs incorporation increased, as fitted in **Figure S2 (e)**. Ref-CIGSSe did not yield a calculated injection barrier E_A since no local maximum $R_{s,diff}$ value was observed; for the Low_Cs-CIGSe device, Cs-CIGSSe, and Overdose_Cs-CIGSSe, the E_A values were determined to be 81.6, 104.8, and 107.2 meV, respectively.

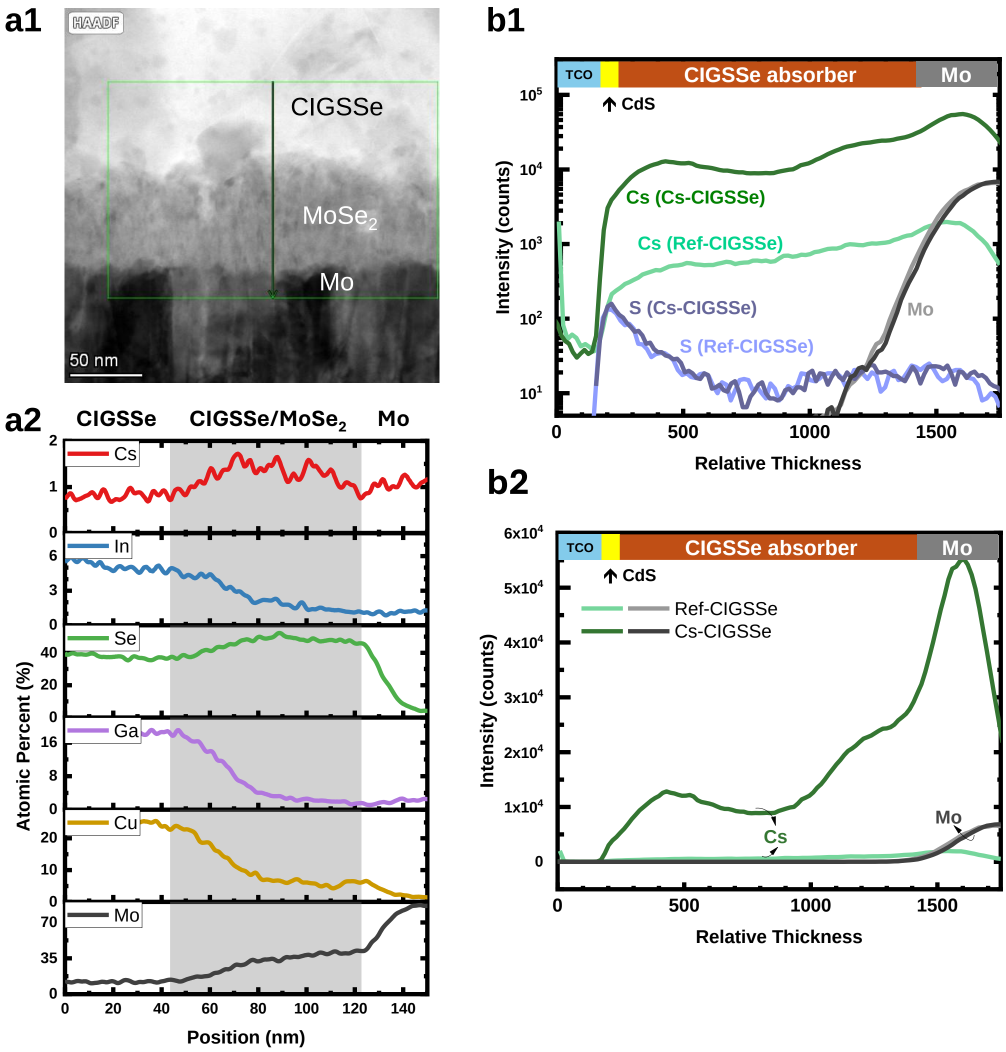

Figure S 4. (a1) TEM high angle annual dark field (HAADF) image at CIGSSe/$MoSe_2$/Mo interface of Cs-CIGSSe device. (a2) EDS scan line through CIGSSe/$MoSe_2$/Mo as indicated in (a1). ToF-SIMS depth profiles of Ref-CIGSSe (light color lines) and Cs-CIGSSe (dark color lines) sample devices presented in (b1) log plot and (b2) linear plot.

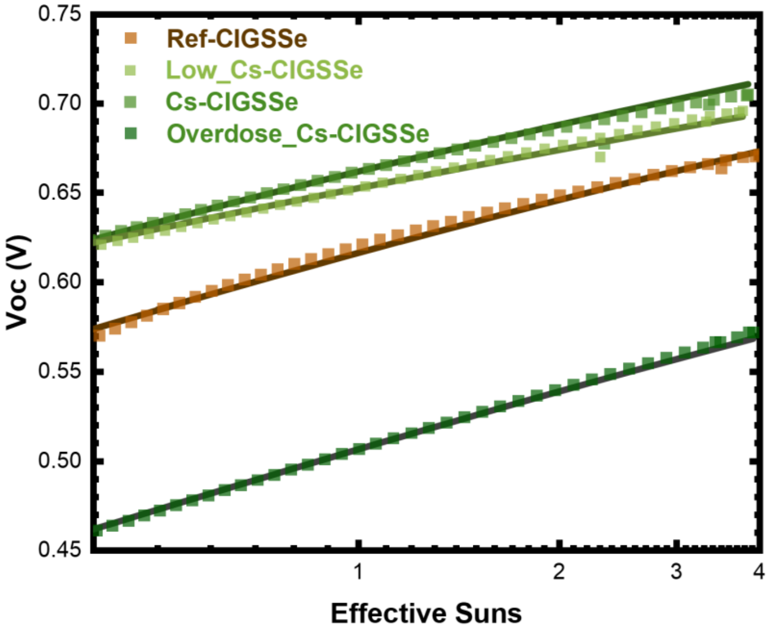

Figure S 5. *Suns-Voc* measurement of the Reference and the Cs-treated devices.

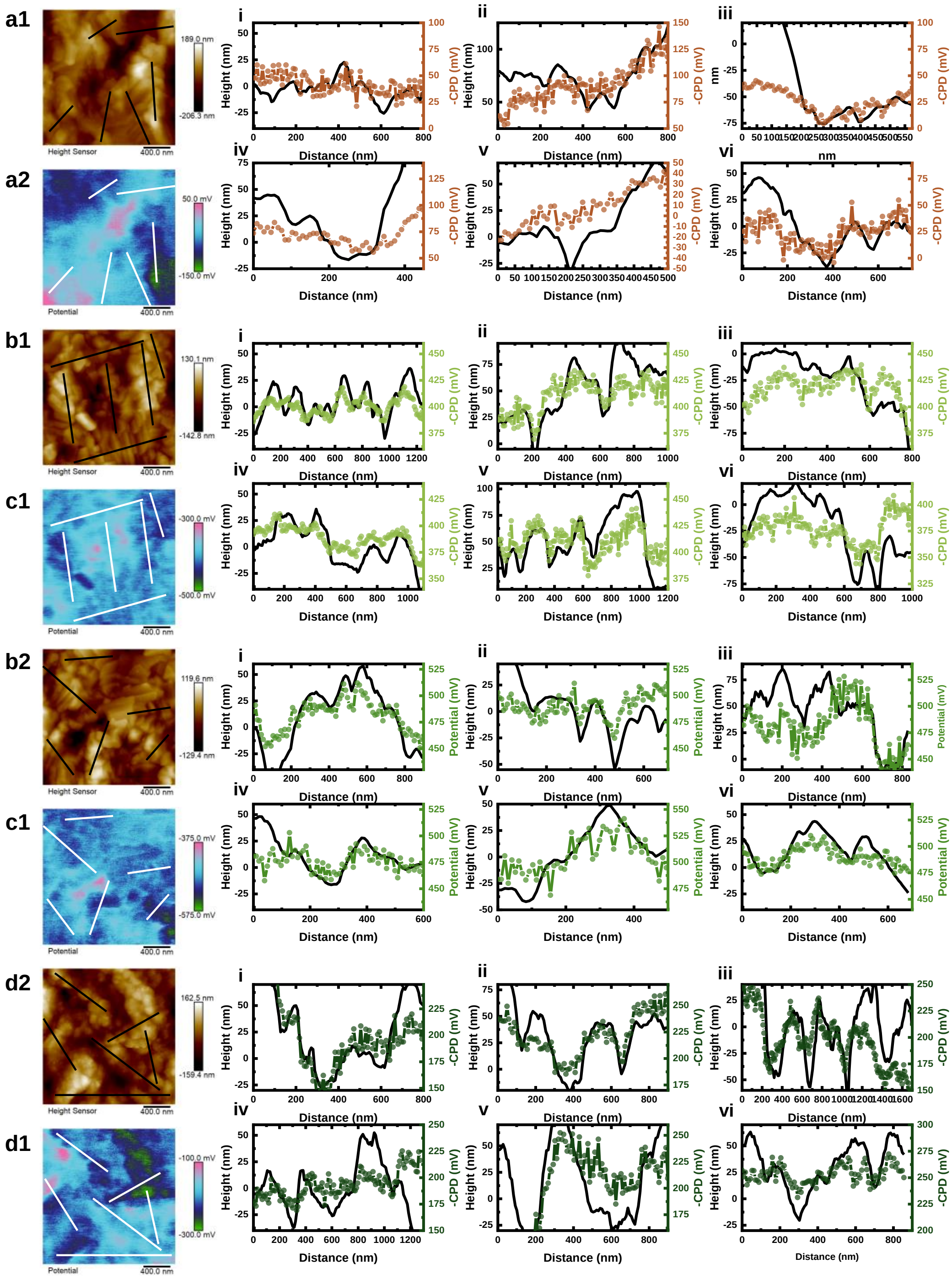

Figure S 6. KPFM measurement of (a1-d1) topography and (a2-d2) contact potential difference (*CPD*). (a1)-(a2) Ref-CIGSSe, (b1)-(b2) Low_Cs-CIGSSe, (c1)-(c2) Cs-CIGSSe and (d1)-(d2) Overdose_Cs-CIGSSe absorber samples.(i)-(vi) Selected six height profiles and corresponding potential profiles across grain and grain boundaries. Note that (i) and (ii) are shown in the main text.

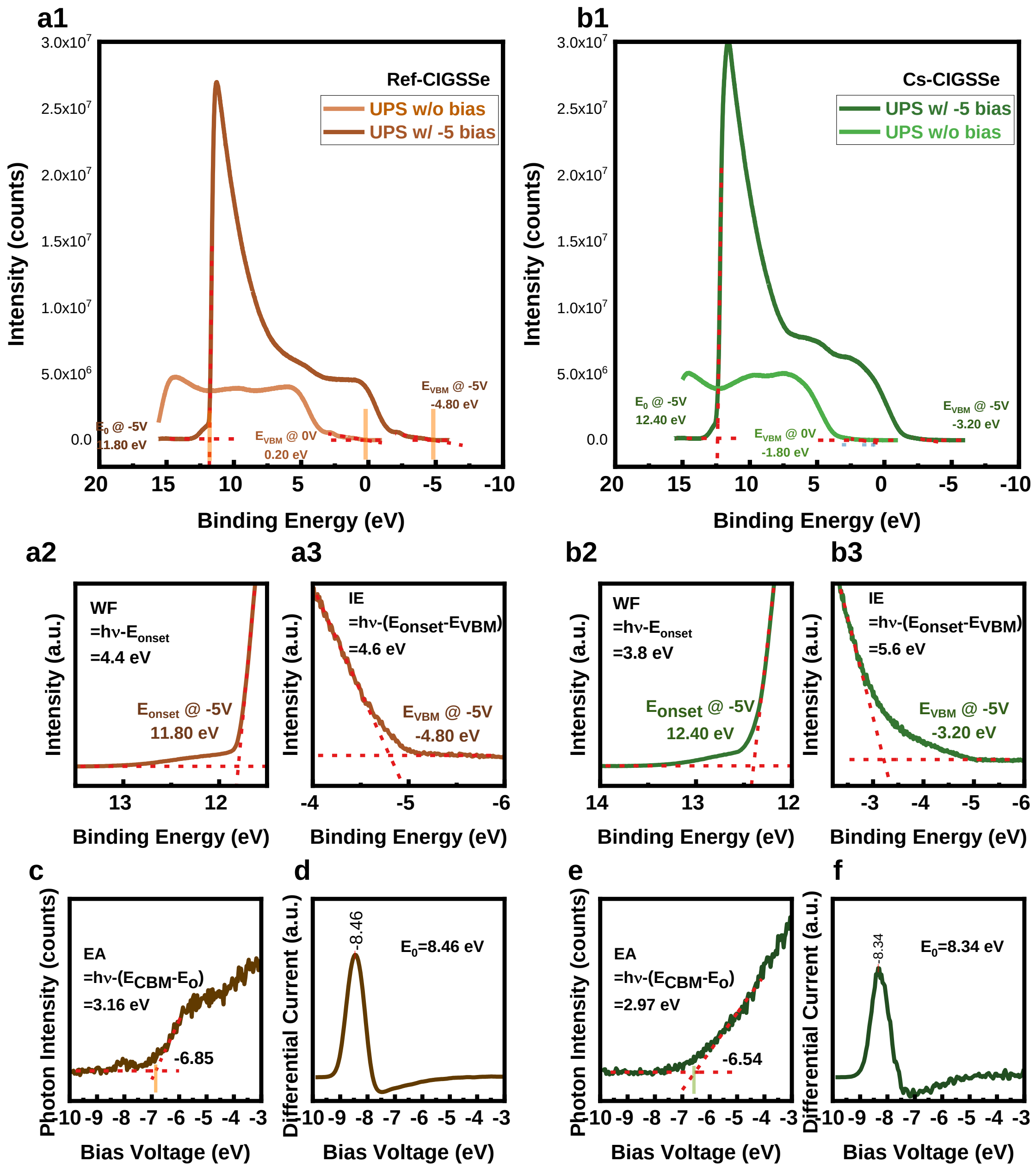

Figure S 7. UPS measurement with -5V bias and without bias of (a1) Ref-CIGSSe and (b1) Cs-CIGSSe samples. Zoom in plot of secondary electron onset energy (E_{onset}) of (a2) Ref-CIGSSe and (b2) Cs-CIGSSe samples. The enlarged plot of VBM @ -5 V of (a3) Ref-CIGSSe and (b3) Cs-CIGSSe samples. (c) LEIPS spectra and (d) differential LEET spectra of Ref-CIGSSe sample. (e) LEIPS spectra and (f) differential LEET spectra of Cs-CIGSSe samples.

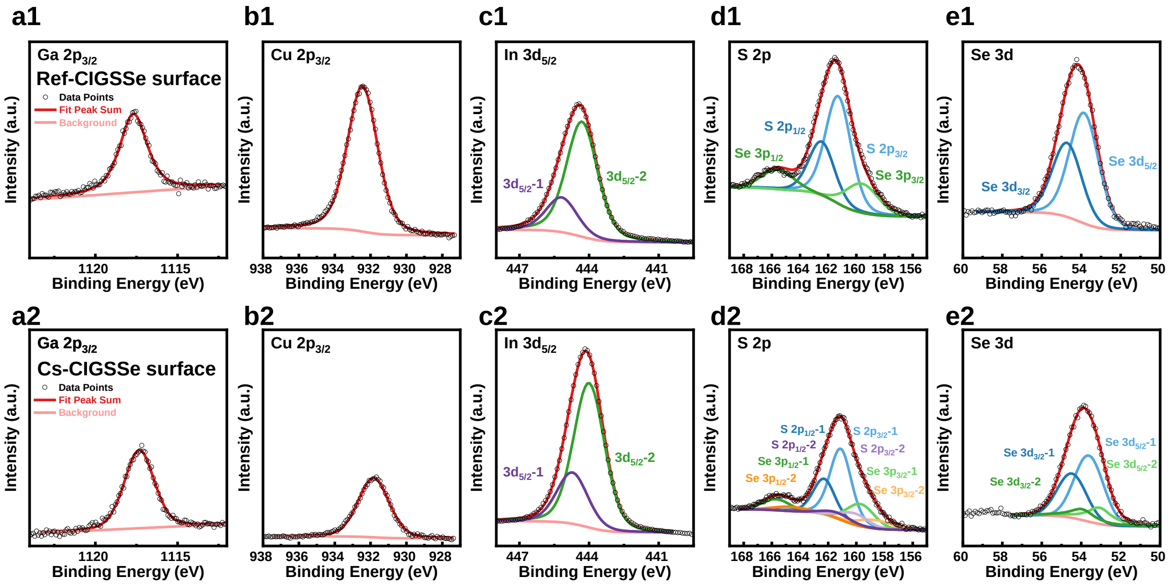

Figure S 8. XPS binding energy profiles of (a) Ga $2p_{3/2}$, (b)Cu $3p_{3/2}$, (c)In $3d_{5/2}$, (d) S 2p, and (e) Se 3d for the front interface of (1) Ref-CIGGSe and (2) Cs-CIGSSe samples.

Table S 3. Atomic percent of CIGSSe at the front surface obtained from XPS measurement and its corresponding calculated bandgap.

Atomic percent	S 2p	Cu $2p_{3/2}$	Ga$2p_{3/2}$	Se3d	In$3d_{5/2}$	GGI	SSSe	E_g (eV)
**Ref-CIGSSe surface**	0.325	0.228	0.048	0.104	0.295	0.141	0.757	1.504

The formula for the E_g of the CIGS-based solar cells containing S is indicated in **Equation S 6** where x and y indicate GGI (Ga/(Ga+In)) ratio and SSe (S/(S+Se)) ratio, respectively [42].

$$Eg_{CIGSSe} = 1 + 0.13x^2 + 0.08x^2y + 0.13xy + 0.55x + 0.54y \qquad \textbf{Equation S 6}$$

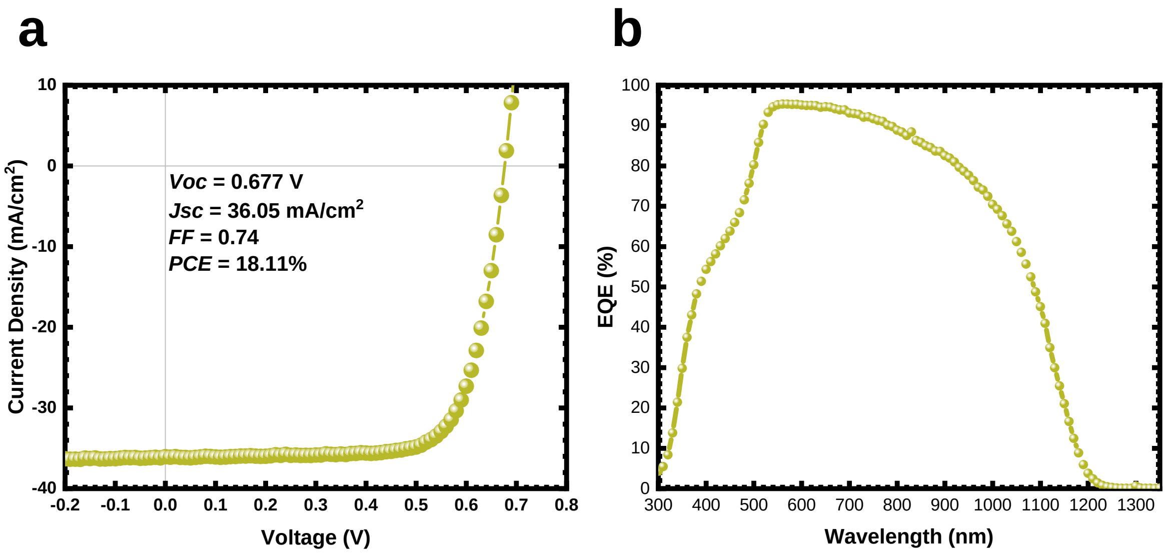

Figure S 9. The (a) *J-V* and (b) *EQE* characteristic curves of Low_Cs-CIGSSe devices with anti-reflection coating, showcasing the champion cell with a device area of 0.27 cm^2.

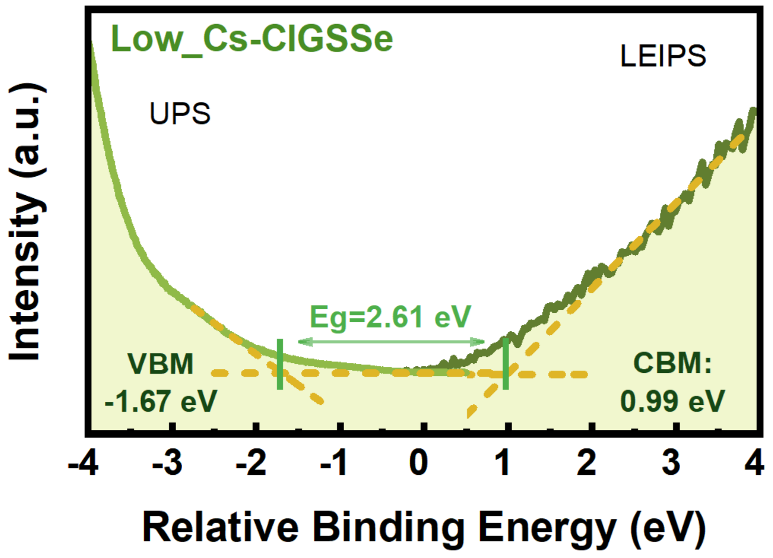

Figure S 10. UPS and LEIPS measurement for front interface band gap of Low_Cs-CIGSSe absorber.
